# An improved strategy for CRISPR/Cas9 gene knockout and subsequent wildtype and mutant gene rescue

**DOI:** 10.1371/journal.pone.0228910

**Published:** 2020-02-13

**Authors:** Jiankang Jin, Yan Xu, Longfei Huo, Lang Ma, Ailing W. Scott, Melissa Pool Pizzi, Yuan Li, Ying Wang, Xiaodan Yao, Shumei Song, Jaffer A. Ajani

**Affiliations:** The University of Texas MD Anderson Cancer Center, Department of Gastrointestinal Medical Oncology, Houston, TX, United States of America; University of Michigan, UNITED STATES

## Abstract

A fluorescence marker mOrange was inserted to the popular pLentiCrispr-V2 to create pLentiCrispr-V2-mOrange (V2mO) that contained both a puromycin selection and a fluorescent marker, making viral production and target transduction visible. Lentiviruses packaged with this plasmid and appropriate guide RNAs (gRNAs) successfully knocked out the genes RhoA, Gli1, and Gal3 in human gastric cancer cell lines. Cas9-gRNA editing efficiency could be estimated directly from Sanger electropherograms of short polymerase chain reaction products around the gRNA regions in Cas9-gRNA transduced cells. Single cloning of transduced target cell pools must be performed to establish stable knockout clones. Rescue of wildtype (RhoA and Gal3) and mutant (RhoA.Y42C) genes into knockout cells was successful only when cDNAs, where gRNAs bind, were modified by three nucleotides while the amino acid sequences remained unchanged. Stringent on-target CRISPR/Cas9 editing was observed in Gal3 gene, but not in RhoA gene since RhoA.Y42C already presented a nucleotide change in gRNA5 binding site. In summary, our improved strategy added these advantages: adding visual marker to the popular lentiviral system, monitoring lentiviral production and transduction efficiencies, cell-sorting Cas9+ cells in target cells by fluorescence-activated cell sorting, direct estimation of gene editing efficiency of target cell pools by short PCR electropherograms around gRNA binding sites, and successful rescue of wildtype and mutant genes in knockout cells, overcoming Cas9 editing by modifying cDNAs.

## Introduction

CRISPR (Clustered Regularly Interspaced Short Palindromic Repeats) is a powerful gene editing tool capable of performing DNA cleavage with the help of guide RNAs (gRNAs) and the constitutive expression of Cas9 [1] [2]. However, substantial numbers of gene knockout (KO) experiments using pLentiCrispr-V1 or pLentiCrispr -V2, or pLenti-Cas9 plus pLenti-Guide-Puro (Version 3)[3, 4] have failed. Factors contributing to the failure include low titers of the lentiviral Cas9, less-than-perfect gRNAs, inefficient selective markers for constitutive expression of Cas9, difficult-to-transfect target cells (e.g., primary cells), and wildtype (WT) or WT-like clones overgrowing and taking over edited cell pools.

The gene knockout (KO) efficiency of CRISPR/Cas9 mainly depends on cellular Non-homologous End Joint (NHEJ) repair to maintain genomic integrity if a complementary donor is not present when Cas9 cleaves both double strands at gRNA binding sites. NHEJ is an error-prone cellular mechanism that repairs ends with mismatch or frameshift (i.e., 1-nt and 2-nt indels) at the break sites of genomic DNA, thus disrupts target gene expression. However, NHEJ repair resulting in in-frame change(s) of the coding DNA sequences (cDNAs), may still be detectable by Western blotting (WB) if the antibodies are designed for the C-terminals of proteins, though detectable by T7 endonuclease assay, a semi-quantitative editing assay method. Our experiments (unpublished) indicated that, in cell pools with lower numbers of gene-edited cells, wildtype (WT) or WT-like cells would overgrow and dominate the cell pools, leading to negative Western blot results and gene KO failure. Therefore, selecting stable KO clones through single cloning or single-cell cloning from transduced pools is critical to ensure KO efficiency.

With insertion of a fluorescent marker mOrange into the lentiviral vector (i.e., CRISPR/Cas9 plasmid), lentiviral production and titer could be visually monitored and estimated because HEK293T cells become brilliant orange colored. According to our observations, generation of high-titer viruses is critical for target cells to be successfully transduced. mOrange fluorescence also indicates Cas9 expression and puromycin resistance, because all three are under the control of the same EF1α promoter. When target cells are transduced with lentiviruses, cells expressing Cas9 and puromycin resistance become visible as well, and moreover, cells can be sorted by flow cytometry, and viral titer can be assayed by fluorescence-activated cell sorting (FACS) instead of the lengthy colony formation method. A few studies have used other fluorescent markers, such as green fluorescent protein (GFP) or red fluorescent protein (RFP), which were inserted to replace the puromycin resistance gene [5–7]. However, puromycin selection for positive clones is an easy, economical, and fast means of selecting target cells. Currently there is no pLentiCrispr vector with selection markers for both puromycin and fluorescence.

Many driver genes in human cancer cells are mutated either actively or silently. Currently, gain- and loss-of-function assays are still critical experiments for clarifying if a new mutant gene is a driver mutation. Two challenges exist: first, when a gene of interest is endogenously expressed in target cells with a high basal level and in all cell lines, it is problematic to investigate gain-of-function of the mutant by directly re-expressing the mutant gene in the target cells. To this end, an empirical method would be to knock out the endogenous expression using CRISPR technology and then re-introduce a mutant(s) into the knockout population. Second, when a gene is knocked out in cell lines, a cascade of downstream molecular and cellular markers is often altered. Oftentimes, researchers or reviewers want to know if rescue of the original gene would reverse the downstream changes in order to authenticate the gene function. In both scenarios, reintroducing a mutant(s) and/or rescuing of a wildtype gene would fail because Cas9-gRNA by nature disrupts introduced genes. Here in this paper, we report how to overcome both hurdles by using CRISPR technologies with modified cDNAs for gene rescue.

## Materials and methods

### Gastric cancer cell lines

Gastric cancer cell lines N87 (CRL-5822), AGS (CRL-1739), KatoIII (HBT-103), Snu1 (CRL-5971) and Snu16 (CRL5974) were ordered from American Type Culture Collection (ATCC) (Manassas, VA). Cell lines Ycc1 (Yonsei Cancer Center, Seoul, South Korea), Ycc2, GT5 (SK-GT5, Memorial Sloan Kettering Cancer Center, New York, NY), MKN45 (CVCL_0434, Cancer Cell Line Encyclopedia Project [CCLE]) were procured earlier and preserved in this research lab.

### Insertion of mOrange into the lentiviral plasmid pLentiCrispr-Cas9-V2 to create V2mO

**Fig 1:**
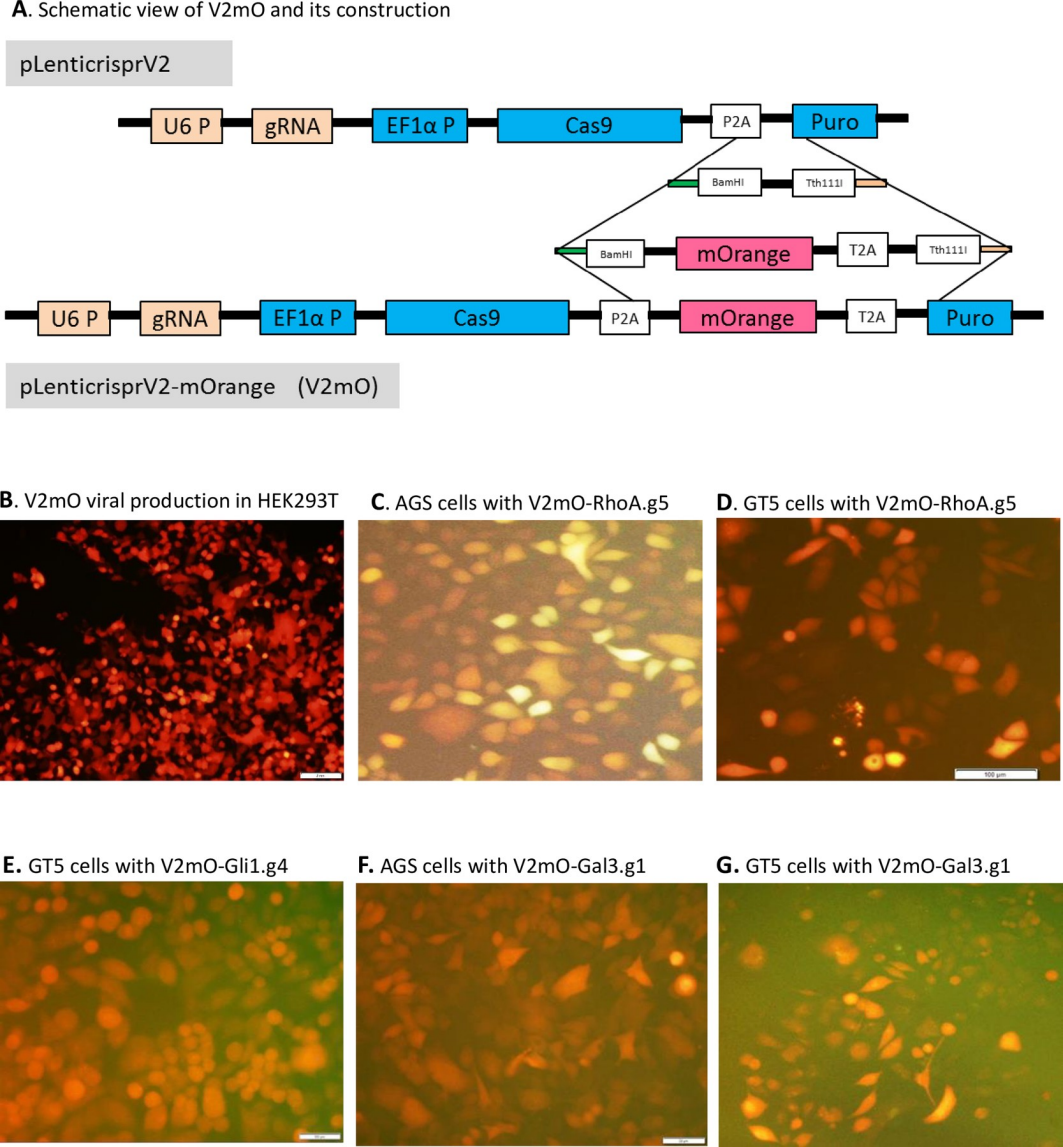
Construction of pLentiCrispr-V2-mOrange (V2mO), its lentiviral production in HEK293T and transduced mOrange expression in GC cell lines AGS and GT5. **A.** V2mO plasmid construct, with mOrange cistronically inserted between Cas9 and puromycin cDNAs. Between the original pLentiCrispr-Cas9-V2 plasmid (top) and the V2mO plasmid (bottom) are shown the overlapping sequences and restriction enzymes for the construction. **B**. HEK293T cells expressed mOrange in lentiviral production, which enables estimation of transformation efficiency and viral titer. **C & D**. Target cell lines AGS (C) and GT5 (D) were transduced with lentiviral V2mO-RhoA.g5 after puromycin selection and mOrange sorting. **E.** Cell line GT5 was transduced with lentiviral V2mO-Gli1.g4 after puromycin selection and mOrange sorting. **F & G**. Cell lines AGS (F) and GT5 (G) were transduced with lentiviral V2mO-Gal3.g1 after puromycin selection and mOrange sorting.

The plasmid pLentiCrispr-Cas9-V2 (Addgene, Cambridge, MA, #52961) is commonly used for gene knockout. In our improved plasmid, a fluorescence gene, mOrange, was amplified with Q5 hifi polymerase (New England Biolabs, Ipswich, MA) with two primers, Crisprv2.mOrange.F and Crisprv2.mOrange.R (Table 1). The forward primer carried a BamHI site and a sequence that overlapped with P2A; the reverse primer carried a Tth111I site, T2A sequence, and a sequence that overlapped the puromycin resistance gene. pLentiCrispr-Cas9-V2 was double-digested with BamHI and Tth111I, and the fragment was gel-purified and ligated with the Q5-PCR product of mOrange using a T4 DNA ligase (New England Biolabs). The resultant plasmids were screened and verified by the double digestion of BamHI and Tth111I, and potential clones were verified by sequencing using hSpCas9.out.F and PuroVar.out.R primers. The end plasmid was V2mO (Fig 1A), which is available from Addgene.

**Table 1:** Primers used in this study.

PCMVfor	5’ CGCAAATGGGCGGTAGGCGTG 3’
T7	5’ TAATACGACTCACTATAGGG 3’
Crisprv2.mOrange.F	5’GATTACAAAGACGATGACGATAAGGGATCCGGCGCAACAAACTTCTCTCTGCTGAAACAAGCCGGAGATGTCGAAGAGAATCCTGGACCGATGGTGAGCAAGGGCGAGGAGAATAACATGGCCATC 3’
Crisprv2.mOrange.R	5’TGGGGACGTCGTCGCGGGTGGCGAGGCGCACCGTGGGCTTGTACTCGGTAGGGCCGGGATTCTCCTCCACGTCACCGCATGTTAGAAGACTTCCTCTGCCCTCCTTGTACAGCTCGTCCATGCCGCCG 3’
hSpCas9.out.F	5’ TGTACGAGACACGGATCGAC 3’
PuroVar.out.R	5’ ACACCTTGCCGATGTCGAG 3’
hRhoA.E3.gRNA5.F	5’ caccgGAACTATGTGGCAGATATCG 3’
hRhoA.E3.gRNA5.R	5’ aaacCGATATCTGCCACATAGTTCc 3’
hRhoA.Dn5Fk.F3.Sac2	5’ aaaCCGCGGaggtggatcggcgtactaga 3’
hRhoA.Dn5Fk.R2.EcoR1	5’ aaaGAATTCagatggcaggatgagaatgg 3’
hRhoA.F.H3.Not1	5’ TGACAAGCTTGCGGCCGCtATGGCTGCCATCCGGAAGAAACTGG 3’
hRhoA.R.Stop.Xba1.BamH1	5’ aaGGATCCTCTAGActaCAAGACAAGGCACCCAGATTTTTTCTTC 3’
hRhoA.Y42C.F2	5’ GTGCCCACAGTGTTTGAGAACTGTGTGGCAGATATCGAGGTGGATG 3’
hRhoA.Y42C.R2	5’ CATCCACCTCGATATCTGCCACAcAGTTCTCAAACACTGTGGGCAC 3’
RhoA.Wt.F.Kpn1	5’ aaGGTACCATGGCTGCCATCCGGAAGAAACTGGTGATTGTTGGTGATG 3’
RhoA.cFlag.R.Apa1	5’ttGGGCCCTTACTTGTCATCGTCATCCTTGTAATCGATGTCATGATCTTTATAATCACCGTCATGGTCTTTGTAGTCCATTCTAGAcaagacaaggcacccagattttttcttcccacg3’
hRhoA.seq.F	5’ cggtctggtcttcagctacc 3’
hRhoA.seq.R	5’ TTAACCGCATAAGGGCTGTG 3’
hGli1.E2.gRNA2.F	5’ caccgGGCTCGCCATAGCTACTGAT 3’
hGli1.E2.gRNA2.R	5’ aaacATCAGTAGCTATGGCGAGCCc 3’
hGli1.E5.gRNA4.F	5’ caccgAGGAAGGCGAGGGCCCTTTT 3’
hGli1.E5.gRNA4.R	5’ aaacAAAAGGGCCCTCGCCTTCCTc 3’
hGli1.seq.g34.F	5’ cagtacttccctgggactgc 3’
hGli1.seq.g34.R	5’ ccagcacccacacctcttta 3’
hGal3.E3.gRNA1.F	5’ caccgCAGACCCAGATAACGCATCA 3’
hGal3.E3.gRNA1.R	5’ aaacTGATGCGTTATCTGGGTCTGc 3’
hGal3.H3Not1.F	5’ aaaAAGCTTGCGGCCGCaatggcagacaatttttcgctc 3’
hGal3.XbaBamH.R	5’ aaaGGATCCTCTAGAttaTATCATGGTATATGAAGCACTG 3’
hGal3.seq.F	5’ tgcctttgccatattcctct 3’
hGal3.seq.R	5’ gataagctccaggtgctcca 3’
hGal3.g12.dg1.F1	5’aaaGCGGCCGCaatggcagacaatttttcgctccatgatgccttatctgggtctggaaacccaaaccctc3’
hGal3.g12.dg2.F2	5’aaaGCGGCCGCaatggcagacaatttttcgctccatgatgccttgtctgggtctggaaacccaaaccctc 3’
hGal3.g12.dg3.F3	5’aaaGCGGCCGCaatggcagacaatttttcgctccatgatgccttgtcagggtctggaaacccaaaccctc 3’

### Guide RNA design, lentivirus production, target cell transduction, puromycin selection and mOrange cell sorting

Our gRNA design followed the methods on Dr. Feng Zhang’s website (*http://crispr.mit.edu*; Massachusetts Institute of Technology; site no longer active). We also referred to German Cancer Research Center’s E-Crisp website (*http://www.e-crisp.org/E-CRISP/designcrispr.html*). Every gene was designed with 3–5 targets of gRNA sequences. Based on previous data, guide sequences from three genes, i.e. RhoA gRNA5, Gli1 gRNA2 and gRNA4, and Gal3 gRNA1, were carried out in this study.

To insert gRNA sequences, duplexes were ligated into V2mO. Briefly, guide RNA forward and reverse primers were allowed to form duplexes in a mix of equal molar concentrations and volumes in a heating block heated from 100°C and cooled gradually on its own. The duplex was then used as an insert ligated using T4 DNA ligase (New England Biolabs) into V2mO precut by BsmbI (New England Biolabs). The ligates were transformed into Stbl3 competent cells and the resultant clones were screened by cracking gel for insert sizes and verified by sequencing. The end lentiviral plasmids were called V2mO-RhoA.g5, V2mO-Gli1.g2, V2mO-Gli1.g4, and V2mO-Gal3.g1.

These four plasmids were then co-transfected with either psPax2 or pCMV.Dr8.2 and with either pMD2.G or pCMV.VSV.G in a ratio of 10:10:1 into HEK293T (ATCC, Manassas, VA) cells with ~70% confluency using Lipofectamine 3000 (ThermoFisher Scientific, Carlsbad, CA) or JetPrime (Polyplus, Illkirch, France). mOrange fluorescence was monitored for transfection efficiency and viral titer estimation. Lentiviral supernatants were harvested in 24 hrs and re-harvested 24 hrs consecutively for 2^nd^ time and 3^rd^ time, lentiviral supernatants were filtered and concentrated by the PEG8000 method into 10 times concentrated. Target AGS and GT5 cells were seeded in 6-well plates with ~70% confluency. Lentiviral supernatants or concentrates were added to the cells with 8 μg/mL polybrene. Transduced cells were then selected by puromycin for 3–6 days using a concentration based on killing curves. Surviving cells were propagated and sorted for mOrange+ cells with a MoFlo flow cytometer in our institutional Flow Cytometry and Cellular Imaging Facility.

### Single cloning, KO verification with Western blot and single clone sequencing

Puromycin-selected and mOrange-sorted pools were subjected to single cloning. The cells were diluted to ~50–100 cells in a 10-cm plate for single cloning. Single clones were propagated and picked in about 1–2 weeks and Western blot was used to verify knockout of RhoA (mAB antibody #2117; Cell Signaling Technology, Danvers, MA, USA), Gli1 (mAB antibody #3538; Cell Signaling Technology) and Gal3 [using an anti-Gal3 antibody made as previously reported [8] [9]). A set of 5 to10 single clones with RhoA or Gal3 knockout were mixed to form KOmixes. To verify if those single clones were truly single or mixed, each clone was subjected to genomic DNA extraction using 50mN NaOH and 1M Tris (pH 7.0), followed by phenol and chloroform purification. PCR products were amplified using Q5 hifi polymerase (New England Biolabs). For the RhoA gene, the primers were hRhoA.Dn5Fk.F3.Sac2 and hRhoA.Dn5Fk.R2.EcoR1. For the Gal3 gene, the primers were hGal3.seq.F and hGal3.seq.R. The PCR products were visualized in 2.0% gel and purified for sequencing. Short electropherograms of the areas near the gRNA binding regions were used for further analyses.

### Short PCR electropherograms for direct estimation of editing efficiency

The initial puromycin-selected and mOrange-sorted pools were harvested and genomic DNAs were extracted, Q5 hifi PCR performed using the method as described above. For the RhoA gene and Gal3 gene, the primers were the same as above. For the Gli1 gene, the primers were hGli1.seq.g34.F and hGli1.seq.g34.R. PCR electropherograms were used directly from Sanger sequencing. For direct estimation of gene editing efficiency, short PCR electropherograms of the areas surrounding the guide RNA regions of cell pools were selected at regions with aberrations. A horizontal line was drawn to average aberrant nucleotide peaks, and then a second line was drawn to average WT nucleotide peaks. Direct estimation of editing efficiency was calculated as Ratio = height of aberration line /(height of aberration line + height of WT line). If the two lines merged at equal height, then the ratio was estimated to be 50% or more.

### Tracking of indels by decomposition (TIDE) and interference of CRISPR edits (ICE) analyses

Tracking of indels by decomposition (TIDE) analyses followed authors’ instruction [10] to identify the major induced mutations in the projected editing sites and determine their frequencies in the cell populations. Briefly, genomic DNAs were extracted from both parental cell lines and CRISPR/Cas9-selected pools of AGS and GT5 pools, then, short PCR products were amplified as described above. PCR primers for RhoA, Gli1, and Gal3 were the same as those used for single clone sequencing and direct estimation of editing efficiency. These analyses were presented as two graphs: the first graph shows the indel spectrum of CRISPR/Cas9-gRNA transduced cell populations compared to parental cell populations, and the second graph shows aberrant sequence signals of CRISPR/Cas9-gRNA transduced cell populations compared to parental cell populations.

Interference of CRISPR edits (ICE) analysis [11] used the same two Sanger sequences as TIDE to analyze indel plots and discordance graphs: one from the control or parental sequence, and the other from the Cas9-gRNA transduced cell population. The PCR primers were the same as those used in TIDE analysis.

### Construction of expressing vectors p3xFlag-RhoA.Wt, -RhoA.Y42C mutant, and -Gal3.Wt wildtype genes

For the RhoA gene, RhoA.Wt cDNA was Q5-PCR-amplified using hRhoA.F.H3.Not1 and hRhoA.R.Stop.Xba1.BamH1, digested with Not1 and BamH1, gel-purified, and ligated into the expression plasmid p3xFlag-CMV-10. Mutant RhoA.Y42C was generated by overlaying two PCR products into a single PCR product: a) product by hRhoA.F.H3.Not1 and hRhoA.Y42C.R2, and b) product by hRhoA.Y42C.F2 and hRhoA.R.Stop.Xba1.BamH1. The expression plasmids p3xFlag-RhoA.Wt and p3xFlag-RhoA.Y42C mutant were digestion-verified and confirmed by sequencing using hRhoA.seq.F and hRhoA.seq.R. For the Gal3 gene, Gal3.Wt cDNA was amplified using hGal3.H3Not1.F and hGal3.XbaBamH.R and subcloned into p3xFlag-CMV-10, and the final plasmid p3xFlag-Gal3.Wt was digestion-verified and sequenced by PCMVfor. Those three plasmids were aligned against GenBank data to make sure no nucleotides were altered, except the intended RhoA.Y42C mutation.

### Construction of rescue plasmids N-terminal Flag p3xFlag-RhoA.dgWt, mutant -RhoA.dgY42C, wildtype -Gal3.dg1, -Gal3.dg2, -Gal3.dg3 and C-terminal Flag p3.1-cFlag-RhoA.dgWt and -RhoA.dgY42C

Because Cas9 cleaves where gRNA binds, one or a few nucleotides in the gRNA5 binding site of the RhoA.Wt, RhoA.Y42C and in the gRNA1 binding site of the Gal3 gene were modified, but without any amino acid being altered. In the RhoA gene, for construction of N-terminal Flag RhoA rescue plasmids, short Q5 PCR products were generated using hRhoA.F.H3.Not1 in combination with hRhoA.dgWt.R for the wildtype and hRhoA.dgY42C.R for the mutant. Clones were screened using digestion by AgeI, BamHI, and EcoRV because modified DNA sequences were absent in the EcoRV site. Positive clones were verified by sequencing. The modified plasmids were called p3xFlag-RhoA.dgWt and p3xFlag-Rhoa.dgY42C; in both of these, three nonconsecutive nucleotides were altered in the gRNA5 binding site, but the amino acid sequences remained unaltered. For construction of C-terminal Flag RhoA rescue plasmids, RhoA.Wt and mutant RhoA.Y42C, three nonconsecutive nucleotides altered RhoA.dgWt and RhoA.dgY42C, were created in the same fashion with primers RhoA.Wt.F.Kpn1 and RhoA.cFlag.R.Apa1. RhoA-Flag PCR products were cloned into pcDNA3.1+. Potential clones were digestion-verified and sequencing-confirmed by T7. The end plasmids were p3.1-cFlag-RhoA.Wt, -RhoA.Y42C, -RhoA.dgWt and -RhoA.dgY42C.

For the Gal3 gene, Q5 PCR paired hGal3.XbaBamH.R with hGal3.g12.dg1.F1, hGal3.g12.dg2.F2, and hGal3.g12.dg3.F3 to generate p3xFlag-Gal3.dg1, -Gal3.dg2, and -Gal3.dg3 plasmids, respectively. Clones were subjected to digestion and sequencing to verify that a single nucleotide, two nonconsecutive nucleotides, and three nonconsecutive nucleotides, respectively, were altered in the cDNAs of gRNA1 binding site of the three plasmids.

## Results and discussion

### The V2mO plasmid with mOrange fluorescent marker expressed in-frame with Cas9 and puromycin gene

To create our improved lentiviral plasmid, mOrange cDNA was integrated cistronically, as EF1α-Cas9-P2A-mOrange-T2A-puromycin, into pLentiCrispr-V2 (Addgene, Watertown, MA; #52961) [3]. The resultant V2mO vector contained three cDNAs of Cas9, mOrange, and puromycin resistance gene, all under the control of a single EF1α promoter. This single polycistronic mRNA could be translated and cleaved, at P2A and T2A self-cleaving peptides, into three functional individual Cas9, mOrange, and puromycin proteins. The integrity of the other components of the original pLentiCrispr-V2 was maintained without any alteration.

Sequencing of potential plasmids using hSpCas9.out.F and PuroVar.out.R verified that two clones were correct (Fig 1A). The original vector, pLentiCrispr-V2 is 14.873 kilobases (kb) in length and, with insertion of the 708-basepair (bp) mOrange, resulted in V2mO of 15.635 kb. With gRNAs inserted, i.e., with the insertion of 20 bp to replace the 1880 bp filler fragment (a nonfunctional fragment for cloning purpose), the V2mO is actually shorter than the popular pLentiCrispr-V2 empty vector (without gRNA insertion). Theoretically, the bigger a plasmid is, the lower its transfection efficiency will be.

To test our improved vector, V2mO was transformed into HEK293T cells with packaging and envelope plasmids. With one gRNA inserted, V2mO transformed competent HEK293T cells with close to 100% fluorescence observed under a fluorescence microscope (Fig 1B). Using the fluorescent marker, we were able to estimate the lentivirus titer in HEK293T cells and the transduction efficiency in target cells. HEK293T cells brightly expressing mOrange not only indicate high-titer viral production, but also add an extra step for target cells’ mOrange sorting by FACS on top of puromycin selection. Our results showed that lentiviral V2mO with gRNAs (e.g., RhoA-gRNA5, Gli1-gRNA4, or Gal3-gRNA1) was also expressed in target cell lines AGS and GT5 (see V2mO-RhoA.g5, Fig 1C and 1D; V2mO-Gli1.g4 in GT5 cells, Fig 1E; and V2mO-Gal3.g1, Fig 1F and 1G), although mOrange expression in target cells was much weaker than in HEK293T cells.

There are a few reports in which puromycin cDNA in the LentiCrispr/Cas9 vector was substituted with a GFP marker, including pSpCas9(BB)-2A-GFP (Addgene #48138) [5] and pL-CRISPR.EFS.GFP (Addgene #57818) [12], and with an RFP marker, including LentiCrispr-RFP (Addgene #75162) [6]. In these studies, GFP and RFP provided good indicators of viral titer and clone selection for target cell screening by flow cytometry. However, puromycin selection is one of the most effective and easiest methods of positive clone selection. Replacement of the puromycin gene by fluorescence may be inconvenient for some laboratories, because they may not have access to a flow cytometer for cell sorting. In contrast, our inclusion of both a puromycin selector and the fluorescent marker mOrange enables inexpensive fast selection and visualization.

### RhoA gene knockout by use of V2mO-RhoA-gRNA5

**Fig 2:**
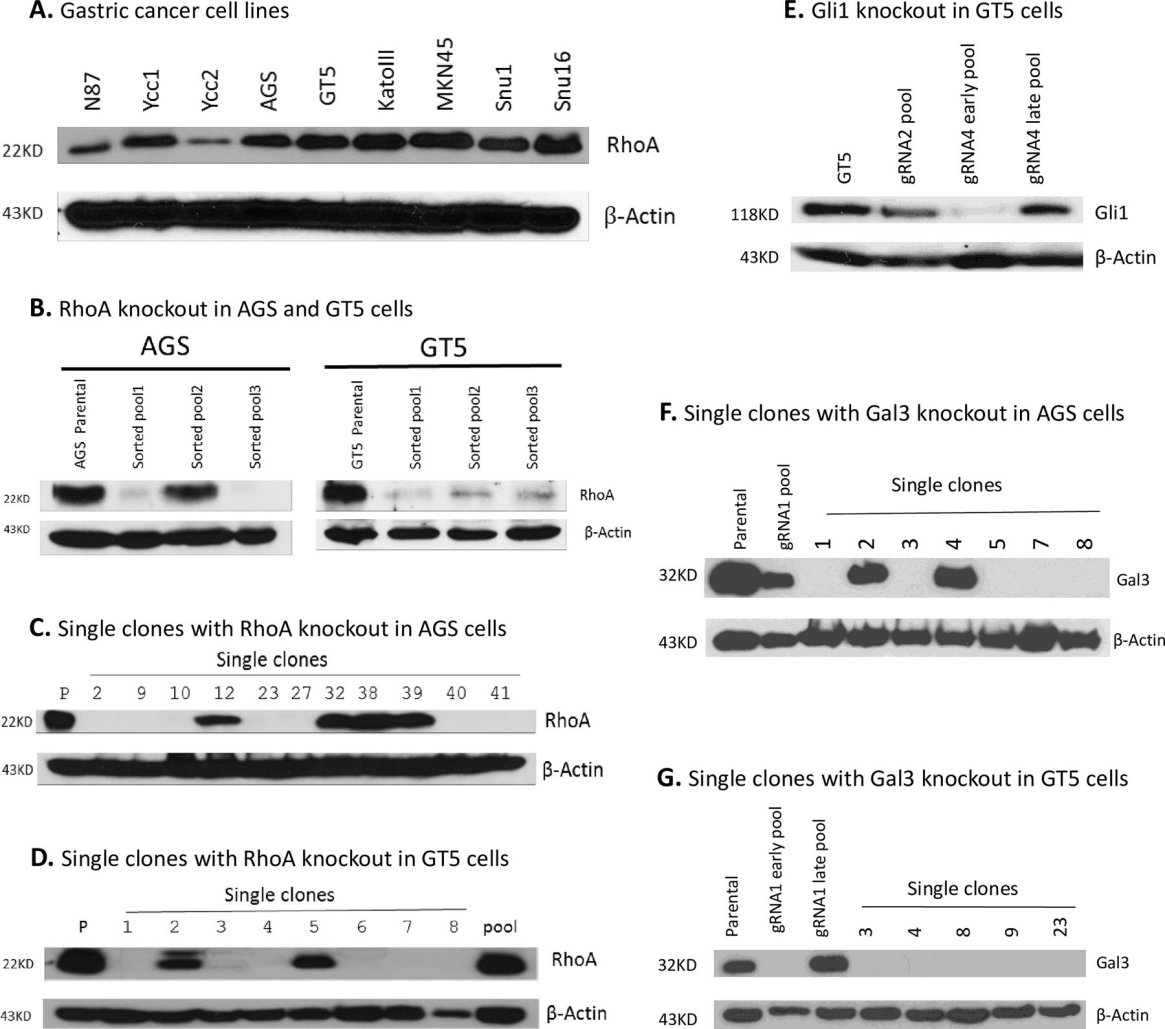
Western blots showing expressions of RhoA, Gli1 and Gal3 in GC cells, their transduced pools and respective single clones isolated from potential pools. **A.** Western blot showed elevated expression of endogenous RhoA in all tested gastric cancer cell lines. **B.** Western blots showed levels of RhoA knockout in cell lines AGS and GT5 transduced with lentiviral V2mO-RhoA.g5 after puromycin selection and mOrange sorting. **C** & **D.** In the AGS and GT5 cell lines, respectively, Western blots of RhoA showed single clones from pool 1 that was transduced with V2mO-RhoA.g5. **E.** Western blots showed Gli1 pools transduced with lentiviral V2mO-Gli1.g2 and g4. **F** & **G.** Western blots of Gal3 in AGS and GT5 cell lines, respectively, and their single clones after transduction with lentiviral V2mO-Gal3.g1.

For RhoA gene, Western blot showed that all of our gastric cancer (GC) lines endogenously expressed RhoA protein (Fig 2A). Sequencing of those cell lines showed that they are all Y42 wildtype (unpublished data); thus, to reintroduce our intended RhoA.Y42C mutant, endogenous expression knockout had to be performed first. After lentiviral transduction of V2mO-RhoA-gRNA5 into the GC cell lines AGS and GT5, puromycin selection, and mOrange sorting, pools of surviving cells in both cell lines showed that the majority of cells were negative for RhoA expression, although AGS cell pool 2 still showed strong RhoA expression (Fig 2B). Of the 11 single clones picked out of AGS pool 1, four remained RhoA positive (Fig 2C). All three GT5 pools showed significantly reduced RhoA expression (Fig 2B). Out of the eight single clones picked out of GT5 pool 1, only two remained RhoA positive, six clones were negative (Fig 2D). Notably, the last GT5 sample which was from the same pool 1 (as in Fig 2B) passed a few passages used to make single cloning, had turned to RhoA positive at the time of Western blot screening. Therefore, it is imperative that single cloning or single-cell cloning from the transduced pools to single out expression-positive clones and obtain stable knockout clones.

RhoA antibody was generated by immunization with a partial C-terminal peptide of RhoA’s 183 amino acids (unpublished communications, Cell Signaling Technology); therefore, Cas9’s editing of genomic RhoA DNA strands with guide gRNA5 was followed by NHEJ repair. Any repair resulting in an indel of triplicate nucleotides would not change the amino acid sequence in the C-terminal and would therefore still be detectable by Western blot, although some of them may have one or two amino acids changed. However, negative clones detected by Western blot indicated that the RhoA gene had been truly disrupted.

To view genome alteration in the vicinity of RhoA gRNA5 binding region, genomic PCR electropherograms were compared. For RhoA KO single clones, PCR electropherograms of genomic DNAs from AGS cell line showed that one clone had an extra A inserted in the 16^th^/17^th^ nucleotide of gRNA5 binding region, the other clone was a two-clone mixture (S1 Fig). In GT5 cell line, one clone KO1 observed an missing A in the 17^th^ nucleotide of gRNA5 binding region, a second clone KO3 saw a mixture of two clones, and the other two clones KO6 and OK8 saw there were 12-nucleotide and 26-nucleotide deletions, respectively, in gRNA5 binding region (S2 Fig).

### Gli1 gene knockout by use of V2mO-Gli1-gRNA4

**Fig 3:**
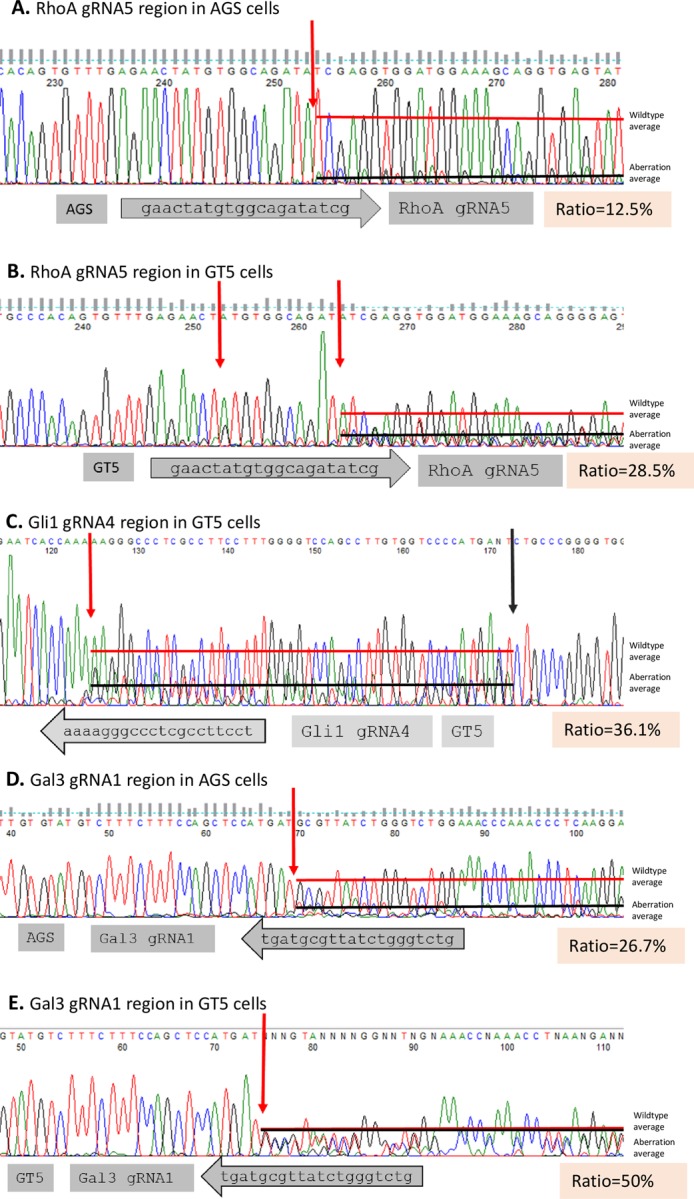
Sequencing electropherograms of PCR products and direct estimation of editing efficiencies around indicated binding sites. **A.** RhoA gRNA5 binding site in AGS cells. Editing efficiency ratio = 12.5%. **B.** RhoA gRNA5 binding site in GT5 cells. Editing efficiency ratio = 28.5%. **C.** Gli1 gRNA4 binding site in GT5 cells. Editing efficiency ratio = 36.1%. **D.** Gal3 gRNA1 binding site in AGS cells. Editing efficiency ratio = 26.7%. **E.** Around the Gal3 gRNA1 binding site in GT5 cells. Editing efficiency ratio = 50%.

For Gli1 knockout, four gRNAs were initially designed. Preliminary tests showed that two, Gli1-gRNA2 and gRNA4, worked. However, in the GT5 cell line, gRNA2 reduced Gli1 expression and Gli1-gRNA4 reduced it substantially indicating that significant numbers of cells had Gli1 knockout (Fig 2E). Puromycin-selected and mOrange-sorted cells were fluorescent in abundant numbers (see Fig 1E), consistent with sequence aberration on the Gli1 PCR electropherogram (Fig 3C). However, it is noted that gRNA4-treated cells that, despite initial Gli1 reduction (early pool), with more cell passages (late pool) Gli1 expression bounced back (Fig 2E), suggesting there were wildtype or wildtype-like cells that increasingly became dominant, rendering Gli1 expression detectable through Western blot.

### Gal3 gene knockout by use of V2mO-Gal3-gRNA1

Our previous experiment showed that Gal3-gRNA1 successfully knocked out Gal3. However, after lentiviral transduction, puromycin selection, and mOrange sorting (Fig 1F), Western blot still picked up Gal3 expression in the original pool of AGS cells, albeit at significantly lowered levels (Fig 2F). Of seven single clones from that pool, five were negative and two were positive for Gal3 expression. In the GT5 cell line, Western blot showed that all five selected clones were negative for Gal3 expression (Fig 2G), which was consistent with our PCR electropherogram data (Fig 3E). It is important to note that, in the GT5 cell line, Western blot of Gal3 revealed that the GT5-gRNA1 late pool with more passages expressed Gal3 protein, whereas the GT5-gRNA1 early pool did not. This strongly suggests that, in a mixed pool of cells transduced with lentivirus and subjected to puromycin selection and mOrange sorting, individual cells were still expressing Gal3 or Gal3 wildtype-like proteins detectable by Gal3 antibody. Based on our findings for the RhoA and Gal3 genes, it is clear that, for a gene to be edited by Cas9, a proper gRNA needs to bind to the binding site; second, in a virus-transduced pool, wildtype or wildtype-like cells may overgrow and dominate over passages, thus, it is necessary to perform single cloning in a mixed cell population.

To view how the genome was altered in the vicinity of Gal3 gRNA1 binding region, genomic PCR electropherograms were compared. For the Gal3 KO single clones, PCR electropherograms of genomic DNAs from AGS cells showed that two clones KO3 and KO5 had an extra T inserted in the 17^th^ nucleotide of gRNA1 binding region. In addition, clones KO1 and KO7 were mixtures of two or more clones, alteration happened even before gRNA1 binding region (S3 Fig). In GT5 cell line, one clone KO23 observed the same extra T inserted in the 17^th^ nucleotide of gRNA1 binding region, the other two clones, KO3 and KO9, were mixtures of two or more clones. Alteration occurred at the 18^th^ and 19^th^ nucleotide, respectively, of gRNA1 binding region (S4 Fig).

### Direct estimation of gene editing efficiency with Sanger electropherograms

We noticed that sequencing Sanger electropherograms of the PCR products flanking the gRNAs gave informative visualization of the gene editing in the cell pools transduced by Cas9-gRNAs. Guide RNAs (gRNAs) were aligned with the Sanger electropherograms, with the gRNA direction pointing to the protospacer adjacent motif (PAM) sequences (NGG). Sequence aberrations, other than noise in the midst of gRNA binding sites before the PAM sequence NGG, were identified, and systemic aberration of those nucleotides compared to wildtype nucleotide peaks was given as direct estimations of the editing efficiencies. Direct estimation of editing efficiency was calculated as Ratio = height of aberration line /(height of aberration line + height of WT line). In the AGS cell line, RhoA gene editing clearly began at the 17th nucleotide of the gRNA5 site (Fig 3A), with an editing efficiency of 12.5% of the cell pool. In the GT5 cell line, RhoA gene editing was detected at the 5th single nucleotide and systemic editing started at the 16th nucleotide (Fig 3B) and afterwards, suggesting random repair by NHEJ in absence of a donor fragment. The estimated editing efficiency was 28.5% in the GT5 cell line. In the same cell line, Gli1 gene editing was detected at the18th nucleotide of the gRNA4 site (Fig 3C), with an editing efficiency of 36.1% of the cell pool. In both the AGS and GT5 cell lines, gene editing of the Gal3 gene was detected at the 16th nucleotide, with editing efficiencies of 26.7% for AGS cells (Fig 3D) and 50% for GT5 cells (Fig 3E).

On the basis of our results for RhoA, Gli1, and Gal3 gene editing, the following observations were made. First, it appears that most gene editing begins between the 16th bp and 18th bp of the gRNA sites, which is consistent with findings in the literature that Cas9 cuts at gRNA’s 17th nucleotide [1] [13]. However, in the absence of donor DNA, cellular repair by NHEJ may randomly produce other forms of editing, such as that we observed at the 5th nucleotide of RhoA gRNA5 in the GT5 cell line. Second, Sanger electropherograms are informative and could be used for direct estimation of gene editing efficiencies of cell pools. Third, because Cas9 is already constitutively expressed as it is integrated into the genome of a cell line, it becomes advantageous that additional gene or genes knockout could be added on top of the gene of interest; only one or more gRNAs in a suitable vector would be required. For example, Lenti-Guide-Puro (Addgene #52963) [3] with an additional gRNA, which also adds higher viral titer, or Lenti-multi-Guide (Addgene #85401) could be used with multiple gRNAs [14].

### Comparison of the editing efficiency by direct electropherogram estimation, TIDE and ICE analyses

**Fig 4:**
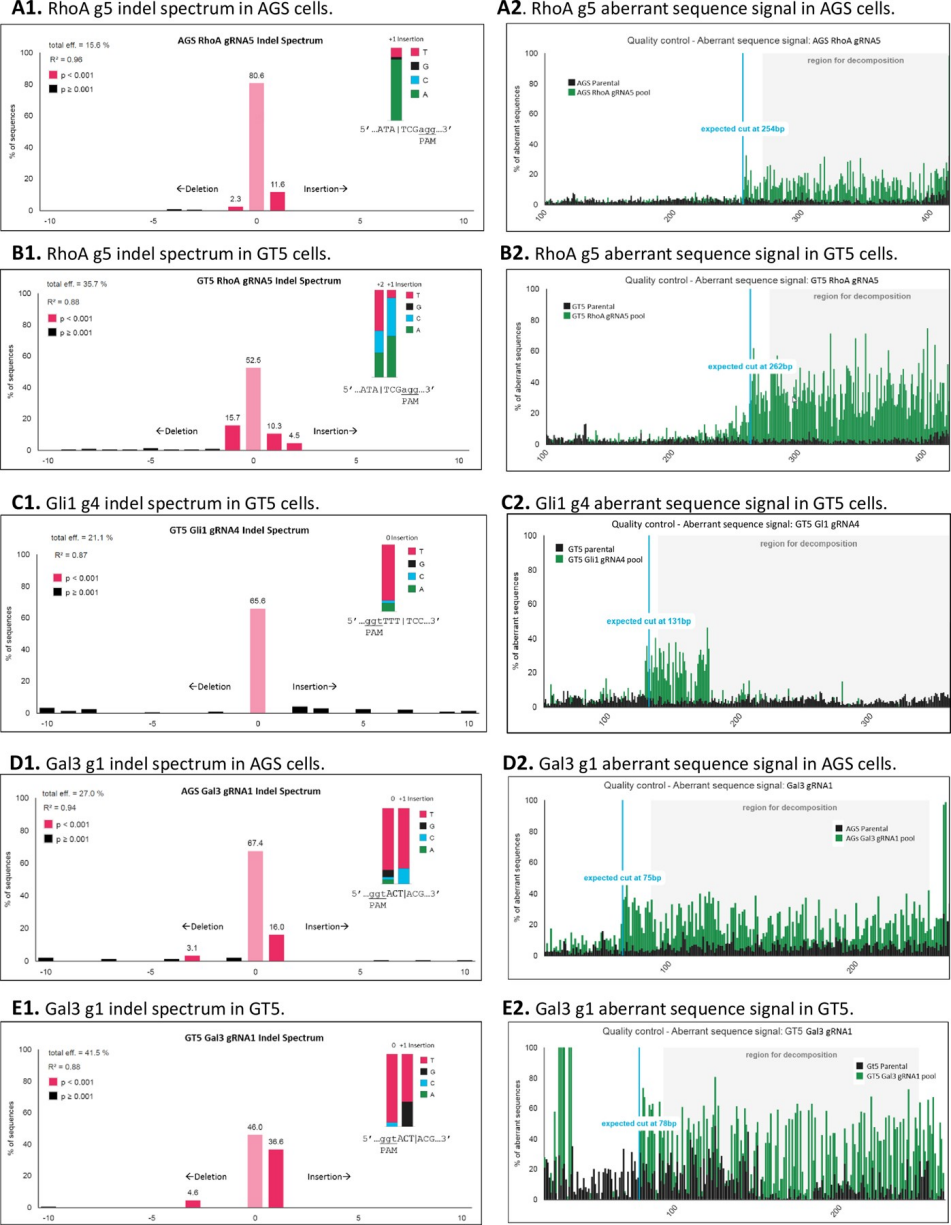
TIDE (tracking of indels by decomposition) analyses showing the indel spectra and aberrant sequences of CRISPR/Cas9-gRNA-transduced cell populations versus untreated parental cell populations. The graphs on the left analyzed indel frequencies within ±10 bp from theoretical gRNA breakpoints. The graphs on the right depicted PCR sequence aberrations; theoretical gRNA cuts were indicated by blue lines. **A1** & **A2.** The RhoA gRNA5 region in AGS cells. Total editing efficiency = 15.6%. **B1** & **B2.** The RhoA gRNA5 region in GT5 cells. Total editing efficiency = 35.7%. **C1** & **C2.** The Gli1 gRNA4 region in GT5 cells. Total editing efficiency = 21.1%. **D1** & **D2.** The Gal3 gRNA1 region in AGS cells. Total editing efficiency = 27.0%. **E1** & **E2.** The Gal3 gRNA1 region in GT5 cells. Total editing efficiency = 41.5%. Eff, efficiency.

TIDE analysis identifies the major induced mutations in a projected editing site and determines their frequency in a cell population. This analysis uses a sophisticated algorithm adopting only two electropherograms: one from a Cas9-gRNA-treated cell population, and the other from an untreated control or parental cell population. In our study, TIDE analysis provided very informative data showing that RhoA-gRNA5 and Gal3-gRNA1 treatment resulted in statistically significant (p<0.001) insertion and/or deletion frequencies (Fig 4A1, 4B1, 4D1 and 4E1, red short columns), with the expected Cas9 cut at the beginning of the aberrant sequences (Fig 4A2, 4B2, 4C2, 4D2 and 4E2). This analysis was consistent with the 16th-18th nucleotide cut detected by direct electropherogram estimations (Fig 3). V2mO-RhoA-gRNA5 gave overall editing efficiencies of 15.6% and 35.7% in the AGS and GT5 cell lines respectively, and V2mO-Gal3- gRNA1 gave overall editing efficiencies of 27.0% and 41.5% in the AGS and GT5 cell lines respectively. The only inconsistency in results between TIDE analysis and direct electropherogram estimation was seen for the GT5 Gli1-gRNA4 cell population, in which direct electropherogram estimation based on gRNA region gave an efficiency of 36.1%, while TIDE analysis gave only 21.1% and a statistically insignificant indel spectrum (p>0.001) (Table 2, Fig 4C1), although direct electropherogram showed abundant sequence aberration in Gli1-gRNA4 region (Fig 3C).

**Table 2:** Comparison of the editing efficiencies by direct electropherogram estimation, and TIDE and ICE analyses.

	Gene targets and cell lines				
	RhoA		Gli1	Gal3	
Method of assessment	AGS	GT5	GT5	AGS	GT5
Direct estimation (%)	12.5	28.5	36.1	26.7	50
TIDE analysis (%)	15.6	35.7	21.1	27.0	41.5
ICE analysis KO score	16	40	17	25	42

**Fig 5:**
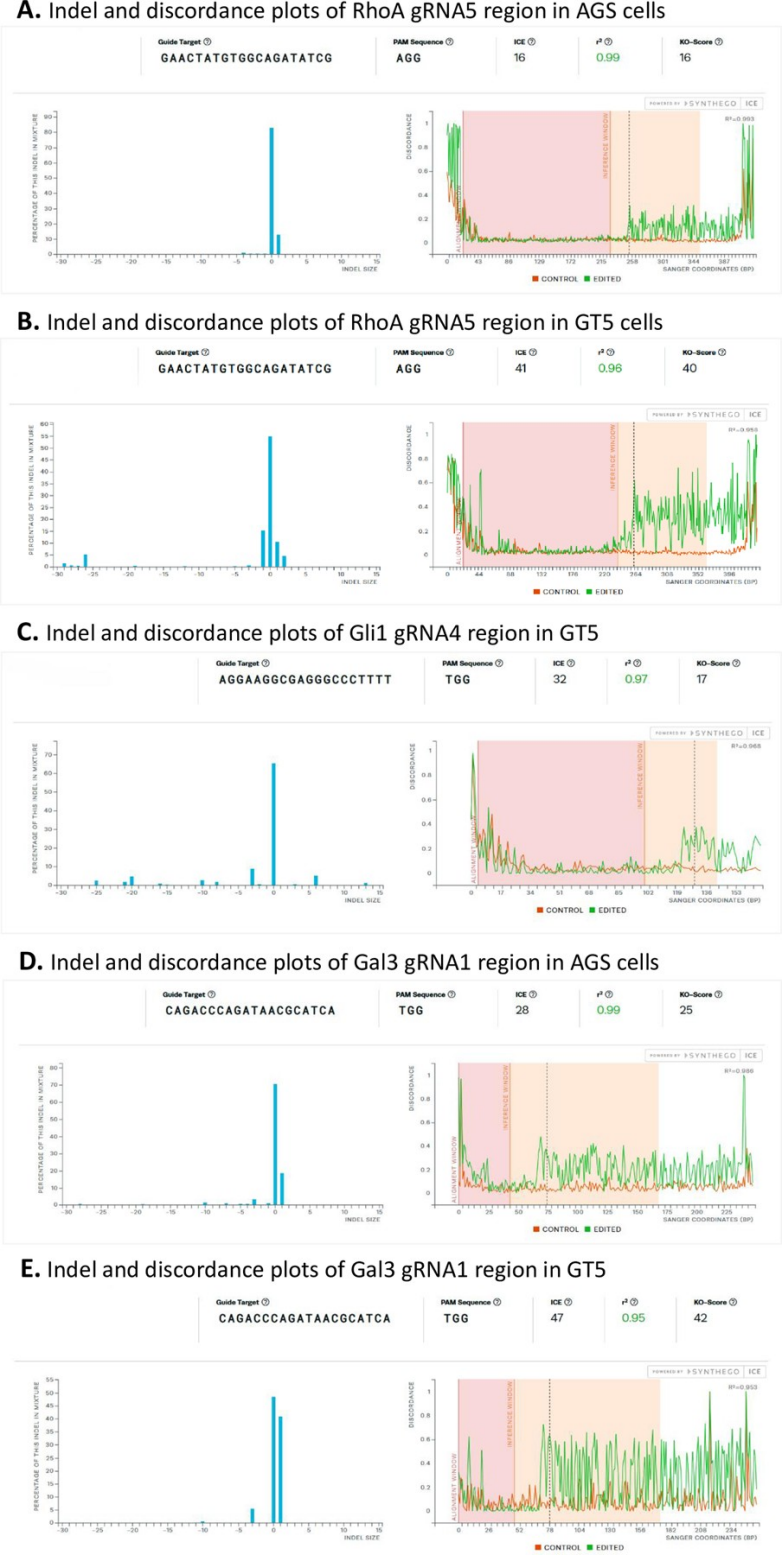
ICE (Inference of CRISPR edits) analyses showing indel and discordance plots. **A.** The RhoA gRNA5 region in AGS cells showing a knockout (KO) score of 16. **B.** The RhoA gRNA5 region in GT5 cells showing a KO score of 40. **C.** The Gli1 gRNA4 region in GT5 cells showing a KO score of 17. **D.** The Gal3 gRNA1 region in AGS cells showing a KO score of 25. **E.** The Gal3 gRNA1 region in GT5 cells showing a KO score of 42.

ICE analysis (*ice*.*synthego*.*com*) [11] provides an alternative means of analyzing CRISPR gene editing efficiency. ICE uses the same two electropherograms that TIDE uses: one from a Cas9-gRNA-treated cell population, and the other from an untreated control or parental cell population. ICE gives an ICE score (an indel percentage), a KO score (proportion of indels that indicate frameshifts), and an r^2^ regression showing the degree of alignment between the treated and control (parental) cell populations (Fig 5). Our r^2^ regression results showed that RhoA-gRNA5 in the AGS and GT5 cell lines, Gli1-gRNA4 in the GT5 cell line, and Gal3-gRNA1 in the AGS and GT5 cell lines all achieved r^2^ scores of 0.95 to 0.99. The KO scores ranged from 16 to 42 and showed similar trends to those seen in the TIDE analyses of the same sequences on a scale of 1 to 100, with 1 being the least efficient and 100 being the most efficient (Fig 5A–5E). ICE analysis gave the GT5 cell line’s Gli1-gRNA4 pool a KO score of 17; these results were similar to those from the TIDE analysis. In the ICE analysis, bad readings on two ends of the Sanger sequences were more pronounced in its discordance graphs than in TIDE analysis, because the latter could adjust parameters to avoid this phenomenon. For the indel percentage, both TIDE and ICE gave very similar patterns and percentage readings for the three genes and two cell lines and thus corroborated each other.

Direct electropherogram estimation assesses ratios of aberrant nucleotides to wildtype nucleotides near gRNAs binding sites from Cas9 cutting sites; this method encompasses short reads that are generally 65–70 nucleotides in lengths. In contrast, TIDE and ICE analyses use longer Sanger electropherograms, normally 300–450 bp. This explains why, in comparing direct electropherogram estimation results to TIDE and ICE analyses results for Gli1-gRNA4 in the GT5 cell line, direct electropherogram estimation showed a higher editing efficiency, while TIDE and ICE analyses showed lower efficiency scores. Because Gli1-gRNA4 in the GT5 cell line altered a region equivalent to 48 bp (16 amino acids) in the parental wildtype GT5 cells (Fig 3C, [between the red and black arrows] and Fig 4C2), which could well harbor indels that could cause a frameshift and thereby disrupt Gli1 expression.

Edit deconvolution by inference of traces in R (EditR) software (*baseEditR.com*) [15] which assesses gene editing using Cas9-Cytidine deaminase fusion enzymes, mainly focuses on gene editing with single-base resolution and without the need for double-stranded break induction. Because the latter is the cellular mechanism on which gene editing or gene knockout is relied upon and intended, therefore, EditR was not included in our editing efficiency comparison.

TIDE analysis uses mostly aberrant sequences of the Cas9-gRNA population to give total efficiency calculations, whereas ICE uses discordance of the Cas9-gRNA population in a similar fashion to give KO scores. In comparison, we propose that direct estimations of Sanger electropherograms surrounding gRNA regions, although simple, are more straightforward and effective for estimating how much of a pool contains Cas9-gRNA-edited or disrupted cells. Moreover, direct electropherogram estimation uses only Cas9-gRNA transduced pools, without the need to sequence control or parental cell lines, because wildtype or wildtype-like cells always exist in substantial proportions in those pools.

### Gene rescue into KO pools requires cDNA modification

Our study of the RhoA gene was originally intended for reintroducing a clinically prevalent mutant Y42C into the gastric cancer (GC) cell lines AGS and GT5. The Catalogue of Somatic Mutations in Cancer (COSMIC) [16] shows that, of 1854 primary stomach cancer samples, 26 samples had RhoA genes with Y42C and Y42S mutations, yielding a prevalent mutation rate of 1.40%, the highest point mutations in the RhoA gene.

**Fig 6:**
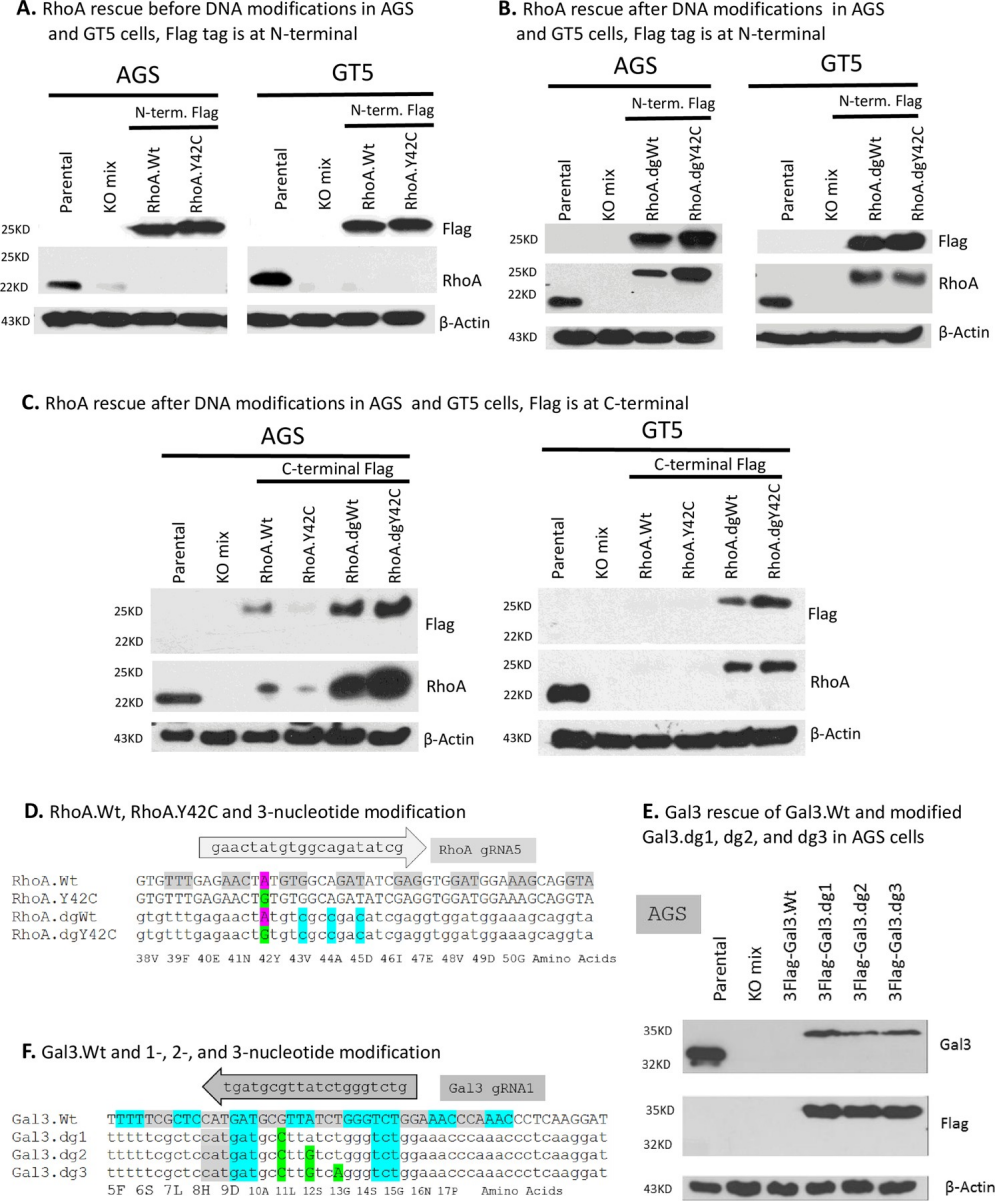
RhoA wildtype, RhoA.Y42C and Gal3 gene rescue and cDNA modifications. **A & B.** Western blots of RhoA, Flag, and β-actin showed before (A) and after (B) three nonconsecutive nucleotide modifications in the gRNA5 binding site. **C.** Western blots of RhoA, Flag, and β-actin showing RhoA rescue after DNA modifications in AGS and GT5 cells with Flag being at C-terminal. **D.** Alignment of rescue plasmids p3xFlag-RhoA.dgWt and p3xFlag-RhoA.dgY42C at the gRNA5 binding site was shown aligned alongside the original RhoA genomic DNA sequence. RhoA gRNA5 was shown at the top, and the amino acid translation was shown at the bottom. Note that wildtype is highlighted in pink for codon Y(TAT), while mutant is Y42C is highlighted in green for codon C(TGT). Three nonconsecutive nucleotides modified are in blue. **E.** Western blot of Gal3 in AGS cells showed the effect of overexpressing p3xFlag-Gal3.Wt and nucleotide modified p3xFlag-Gal3.dg1, dg2 and dg3. **F.** Alignment of the rescue plasmids p3xFlag-Gal3.Wt and p3xFlag-Gal3.dg1, dg2, and dg3 with, respectively, 1, 2, and 3 nonconsecutive nucleotides modified from the gRNA5 binding site. The amino acid translations are shown at the bottom. Note that the changed nucleotides are in green. KO, knockout.

One difficulty of studying the Y42C mutant is that RhoA is highly expressed in all of the tested GC cell lines (Fig 2A), but sequencing (unpublished data) showed that these GC cell lines do not have a Y42C point mutation. When the plasmids N-terminal Flag-tagged p3xFlag-RhoA.Wt and p3xFlag-RhoA.Y42C were transfected into RhoA KOmixes of AGS and GT5 cells, we did not see RhoA expression detectable by Western blots (Fig 6A), although Flag tag was strongly expressed. Because Flag was fused with RhoA in its N-terminal, this finding strongly suggested that RhoA cDNA was edited by integrated Cas9, rendering RhoA dysfunctional, as undetected by Western blot, although p3xFlag-RhoA.Y42C already presented a nucleotide change in the gRNA5 binding site (Fig 6D).

Re-overexpressing N-terminal Flag-tagged p3xFlag-RhoA.dgWt and p3xFlag-RhoA.dgY42C with an extra three nonconsecutive nucleotides modified in the gRNA5 binding site (Fig 6D) into the same RhoA KOmixes of AGS and GT5 cells brought back expression of both Flag and RhoA (Fig 6B), which strongly suggested that Cas9 was still cleaving any unmodified, reintroduced RhoA wildtype and Y42C mutant; therefore modifying cDNA sequence within gRNA binding sites becomes a requirement (Fig 6B, 6C and 6E). Native RhoA protein is estimated to be 22 kDa in size; the addition of a 3xFlag of 81 nt (27 amino acids) added 27 x 110 Da, or 2970 Da, to the RhoA protein, making it an estimated 25 kDa in size.

To address why Flag tag was expressed but RhoA was not in AGS and GT5 RhoA KOmixes when reintroduced RhoA cDNAs were not modified, RhoA cDNAs with C-terminal Flag, p3.1-cFlag-RhoA.Wt, -RhoA.Y42C, -RhoA.dgWt and -RhoA.dgY42C, were constructed without and with three nonconsecutive nucleotides modification in the gRNA5 binding region. Western blots showed that both RhoA and Flag expressed strongly in transiently transfected RhoA KOmixes of AGS and GT5 only with three nonconsecutive nucleotides modification in gRNA5 binding region (Fig 6C). This finding strongly corroborated our assumption that Cas9 was editing reintroduced RhoA cDNA if gRNA binding region was not modified. The remnant bands of Flag and RhoA in AGS by unmodified RhoA probably indicated that introduced RhoA cDNA copies were more than integrated Cas9’s editing capability. Therefore to highly overexpress RhoA protein, three-nonconsecutive modification of RhoA in gRNA5 binding region is required.

Rescue of wildtype Gal3 expression by transfecting p3xFlag-Gal3.Wt into the AGS Gal3-KOmix cell pool also yielded dysfunctional expression undetectable by Western blot (Fig 6E). Thus, p3xFlag-Gal3.dg1, Gal3.dg2, and Gal3.dg3 with 1, 2, and 3 nonconsecutive nucleotides modified at the gRNA5 binding site (Fig 6F) were transfected into the Gal3-KOmix pool. Western blot showed that all produced the expected 35 kDa Gal3 protein and Flag tag (Fig 6E). However, the Western bands of Gal3 were markedly weaker than those for the parental cell population, perhaps because the inserted 3xFlag tag in the N-terminal of the Gal3 altering peptide epitope structure caused reduced sensitivity to our Gal3 antibody. Thus, in the case of the Gal3 gene, CRISPR/Cas9 is very stringent in its on-target editing and avoids off-target editing. Combining this finding with that for the RhoA gene, 3-nucleotide modification in the middle of gRNA binding sites seemingly would ensure the successful rescue of wildtype and mutant gene expression by exogenous introduction.

### Off-target editing by CRISPR/Cas9 observed in RhoA gene but not in Gal3 gene

Off-target genome editing refers to nonspecific and unintended genetic modifications, which consist of unintended point mutations, indels, inversions, and translocations [17–21]. In this study, CRISPR/Cas9 editing on DNAs other than gRNA-matched sequences is considered off-target editing. In the Gal3 gene, CRISPR/Cas9 was very stringent in on-target editing around the gRNA1 binding site, as observed by short DNA sequencing in which only one nucleotide modification of gRNA1 binding site prevented Gal3 from being edited (Fig 6E and 6F), while straight transfection of wildtype p3xFlag-Gal3.Wt without any cDNA modification led to detection of neither Gal3 nor Flag tag expression by Western blots (Fig 6E). In comparison with the rescue plasmid RhoA.Wt and the mutant plasmid RhoA.Y42C, Y42C already presents a nucleotide change in the binding site of RhoA gRNA5, yet, as shown in Fig 6A, reintroduced p3xFlag-RhoA.Y42C did not result in detection of RhoA expression in transiently expressed pool, suggesting an evidence of CRISPR/Cas9 off-target editing, an obvious disadvantage of the CRISPR/Cas9 system.

It has been reported that high frequency off-target activity by presented Cas9-gRNA could rise to >50%, which will be a big concern [22]. It was identified that an active Cas9 can tolerate mismatches of gRNAs with targets harboring up to five mismatches, which has important implications in research and therapeutic applications [23]. It has also been shown that 15 off-target sites from 27 different single guide RNAs (sgRNAs), each harboring a single-base bulge and one to three mismatches to the guide strand, showed a significant variety of off-target sites cleaved by Cas9 [20]. But it was argued that by ChIP-seq that inactivated Cas9 binds to many sites of the genome, but activated Cas9 rarely cleaves off-target sites without matching gRNAs [1]. To reduce off-target editing, an optimized U6:3 RNA promoter produces the most potent effects of germline and somatic genome engineering in *Drosophila* in combination with homologous direct repair (HDR) and offsets nicking-based mutagenesis [24]. Another finding is that truncated gRNAs, shorter than 20 nucleotides in length at the far end of the PAM sequences, can decrease undesired mutagenesis at some off-target sites by ≥5,000-fold without sacrificing on-target genome-editing efficiencies [25]. However, in comparison with the major zinc finger nuclease (ZFN) and transcription activator-like effector nuclease (TALEN) gene editing methods, and CRISPR/Cas9 produces the least off-target editing [26]. Elevated Cas9 expression is among those factors affecting off-target effects in the CRISPR/Cas9 method. Therefore, strategies to control Cas9 activation, such as Tet-inducible Cas9, 4-hydroxytamoxifen (4-HT)-inducible Cas9 constructed by fusing a hormone-binding domain of the estrogen receptor (ERT2) to Cas9, or light-activated Cas9 constructed by fusing a light-responsive element to Cas9, have been applied, but these have limitations and drawbacks, such as adding significant complexity for research laboratories. Other simple but efficient approaches, such as using a Cas9 self-targeting system or substituting four amino acids in Cas9, rendering it a “high-fidelity” Cas9 nuclease, are among the best strategies for overcoming off-target effects. SpCas9-HF1, a high-fidelity variant, rendered all or nearly all off-target events undetectable by genome-wide break capture and targeted sequencing methods, thus providing an alternative to wildtype SpCas9 for research and therapeutic applications [27]. By using structure-guided engineering to improve the specificity of *Streptococcus pyogenes* Cas9 (SpCas9), a specificity-enhanced variant, eSpCas9, was developed. This variant displayed reduced off-target cleavage while maintaining robust on-target activity; thus, it could be broadly useful for genome-editing applications requiring a high level of specificity [28]. By using single-molecule Förster resonance energy transfer experiments, a non-catalytic domain REC3 within Cas9 was found and a new hyper-accurate Cas9 variant, HypaCas9, was developed which demonstrated high genome-wide specificity without compromising on-target activity in human cells [29]. In Cas9, a single point mutation (p.R691A) was identified that HiFi-Cas9-R691A reduces global off-target editing while maintaining high on-target activity [30]. Compared to the rationally designed eSpCas9 [28], SpCas9-HF1 [27], and HypaCas9 [29], HiFi-Cas9-R691A demonstrated the clinical utility of HiFi-Cas9 for therapeutic genome-editing applications. An engineered SaCas9-HF Cas9 variant from *Staphylococcus aureus* showed high genome-wide targeting accuracy without compromising on-target efficiency [31].

Our finding of a discrepancy of off-target editing in the RhoA.Y42C-gRNA5 region but not in the Gal3-gRNA1 region might be attributed to the positioning of the one nucleotide mismatch on the gRNAs; the one-nucleotide change in RhoA.Y42C was on the far end of the gRNA5 PAM sequence, whereas the one-nucleotide change in Gal3.Wt.dg1 was closer to the PAM sequence. According to literature, single and double mismatches are tolerated to varying degrees, depending on their positions along the gRNA-DNA interface [23] [25]. Even a target sequence truncated by one or two nucleotides at the very far end of the gRNA PAM sequence was still edited. The closer to the PAM sequence a mismatch is, the less likely it is to be edited. Therefore, raising stringency and specificity, eliminating off-target editing by CRISPR/Cas9 technologies through molecular engineering, and/or discovering novel Cas9-like genes from eubacteria or archaea, such as a high specificity FnCas9 from the bacterium *Francisella novicida* [32], will be the focus for future gene editing research before CRISPR technology could be adopted for human genetic and medical applications.

## Conclusions

Our V2mO lentiviral vector successfully expressed mOrange in-frame with Cas9 and puromycin cDNAs, which added visualization of viral production and estimation of titers, as well as the capability of cell sorting Cas9+ cells in target cells by FACS, while the other components of the original pLentiCrispr-V2 were maintained without any alteration. Our results indicate that generating high titers of viruses is the first step of successful gene editing or gene knockout. When V2mO was made into lentiviruses, with proper gRNAs, i.e. RhoA-gRNA5, Gli1-gRNA4, and Gal3-gRNA1, they sufficiently knocked out RhoA, Gli1, and Gal3 genes in GC cell lines AGS and GT5 as visualized by short PCR electropherograms around gRNA binding regions and detected by Western blots. Gene editing efficiencies or knockout ratios of target cell pools could be estimated by direct Sanger electropherograms from Cas9-gRNAs transduced cell populations without the sequences of control or parental populations. Analysis software, such as TIDE and ICE, was shown to provide very informative data, including gene editing efficiencies, indel frequencies, and aberrant or discordant sequences against sequences of control or parental populations. Western blots also verified RhoA and Gal3 knockout in both AGS and GT5 cell lines. Single cloning of knocked-out cell pools must be performed to establish stable knockout clones; otherwise, pools of transduced cell lines will be gradually overgrown and dominated by wildtype or wildtype-like cells. This study also proved that rescue and re-overexpression of wildtype and mutant genes into knockout pools require cDNA modification by an extra three nonconsecutive nucleotide changes in the gRNA binding sites without alteration of amino acids, with the changes preferably in the midst of the gRNA binding sites or closer to PAM sequences. We have shown that cDNAs reintroduced into knockout populations without sequence modification in the gRNA binding regions will be edited by Cas9. Cas9’s stringent on-target effect was observed in the Gal3 gene, but off-target editing was observed in the RhoA gene because the RhoA.Y42C mutant already presented a nucleotide change in the gRNA5 binding site. Although we successfully rescued and re-overexpressed RhoA.Wt, RhoA.Y42C, and Gal3.Wt back into knockout cell mixes, our findings and other researchers’ publications suggest that, while CRISPR/Cas9 is a powerful method of gene editing, but that off-target or mismatched editing of target sequences is a big concern, particularly for critical medical or clinical applications.

## Supporting information

S1 Fig Electropherograms of single clones of RhoA knockout (KO) from the AGS cell line aligned with wildtype sequence. For each electropherogram, the wildtype (WT) sequence is aligned at the bottom along with gRNA sequence. In AGS cell line, RhoA clones KO2 and KO9 showed genomic sequences in the vicinity of gRNA5 region. Clone KO9 had an extra A inserted at the 16^th^/17^th^ nt, causing frameshift. Clone KO2 was not a single clone, but a mixed one, though Western blot showed it was truly RhoA KO (see Fig 2C).
(DOCX)

S2 Fig Electropherograms of single clones of RhoA knockout (KO) from the GT5 cell line aligned with wildtype sequence. For each electropherogram, the wildtype (WT) sequence is aligned at the bottom along with gRNA sequence. In cell line GT5, four RhoA clones KO1, 3, 6 and 8 showed genomic sequences in the vicinity of gRNA5 region. Various alterations were observed at gRNA5 binding region. Clone KO1 had an A missing at the 17^th^ nt of gRNA5, causing frameshift. Clone 3 was not a single clone, but a mixed one, possibly comprised of 2 clones, though Western blot showed it was truly RhoA KO (see Fig 2D). Clones KO6 and KO8 had 12 nt and 26 nt deleted at the 16^th^ or -2^nd^ nt of gRNA5 binding regions, respectively. Western blot showed both were truly RhoA KO (see Fig 2D).
(DOCX)

S3 Fig Electropherograms of single clones of Gal3 knockout (KO) from the AGS cell line aligned with wildtype sequence. For each electropherogram, the wildtype (WT) sequence is aligned at the bottom along with gRNA sequence. In AGS cell line, Gal3 clones KO1, 3, 5, and 7 showed genomic sequences in the vicinity of gRNA1 region. Clones KO3 and KO5 had an extra T inserted at the 17^th^ nt, causing frameshift. Clones KO1 and KO7 were not single clones, but mixed ones, though Western blot showed that they were truly RhoA KOs (see Fig 2F).
(DOCX)

S4 Fig Electropherograms of single clones of Gal3 knockout (KO) from the GT5 cell line aligned with wildtype sequence. For each electropherogram, the wildtype (WT) sequence is aligned at the bottom along with gRNA sequence. In cell line GT5, Gal3 clones KO3, 9, and 23 showed genomic sequences in the vicinity of gRNA1 region. Clone KO23 had an extra T/C inserted at the 17^th^ nt, causing frameshift. Clones KO3 and KO9 were not single clone, but a mixed one, aberration began at 18^th^ or 19 nt of gRNA binding sequence, though Western blot showed it was truly RhoA KO (see Fig 2G).
(DOCX)
