# Supplementary material for: An improved strategy for CRISPR/Cas9 gene knockout and subsequent wildtype and mutant gene rescue
Source: PLoS One. 2020 Feb 13;15(2):e0228910. doi: 10.1371/journal.pone.0228910 (PMC7018052; doi:10.1371/journal.pone.0228910)
Supplement: S1 Fig — For each electropherogram, the wildtype (WT) sequence is aligned at the bottom along with gRNA sequence. In AGS cell line, RhoA clones KO2 and KO9 showed genomic sequences in the vicinity of gRNA5 region. Clone KO9 had an extra A inserted at the 16th/17th nt, causing frameshift. Clone KO2 was not a single clone, but a mixed one, though Western blot showed it was truly RhoA KO (see Fig 2C). (DOCX) [file pone.0228910.s001.docx]

AGS RhoA Knockout (KO) Single Clones


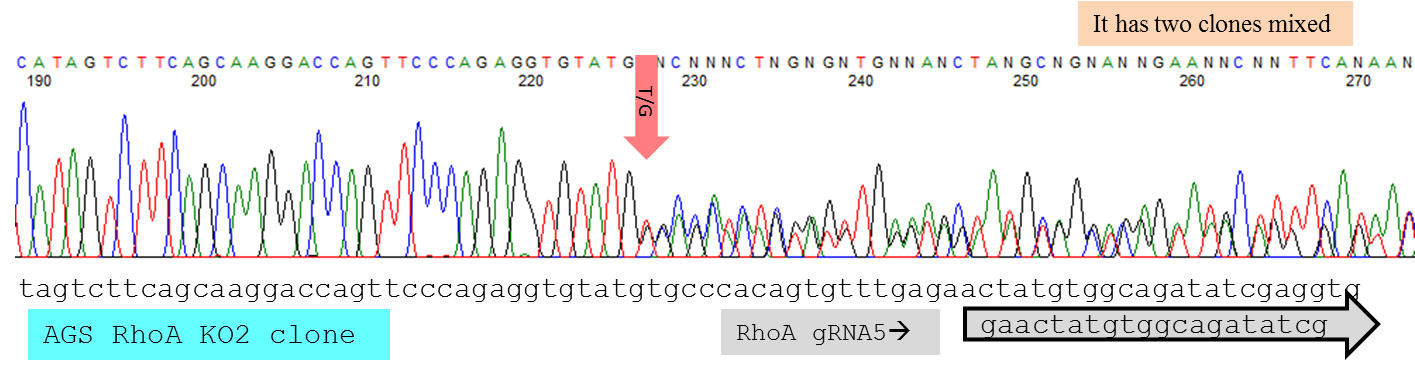


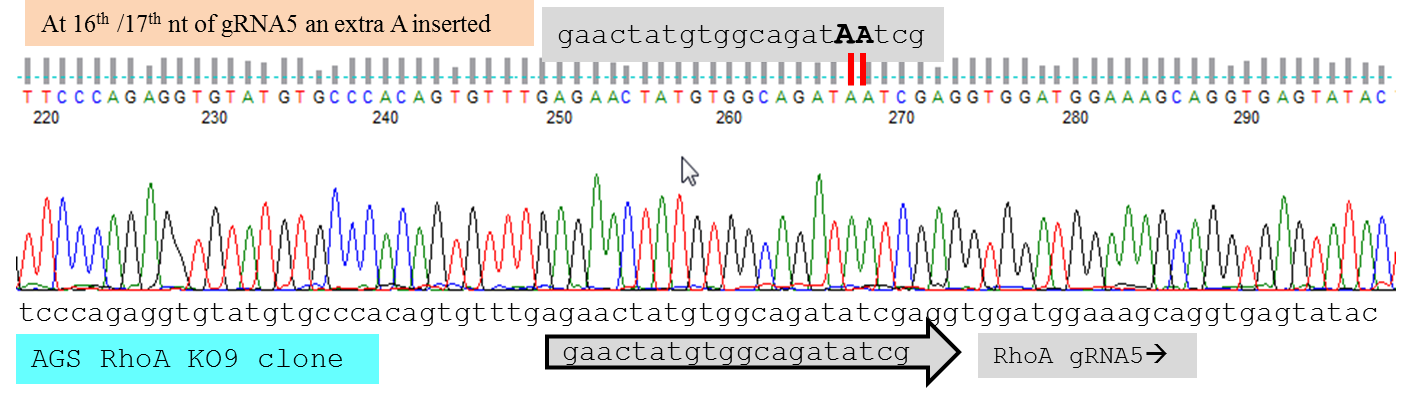


S1 Fig. Electropherograms of single clones of RhoA knockout (KO) from the AGS cell line aligned with wildtype sequence. For each electropherogram, the wildtype (WT) sequence is aligned at the bottom along with gRNA sequence. In AGS cell line, RhoA clones KO2 and KO9 showed genomic sequences in the vicinity of gRNA5 region. Clone KO9 had an extra A inserted at the 16^th^/17^th^ nt, causing frameshift. Clone KO2 was not a single clone, but a mixed one, though Western blot showed it was truly RhoA KO (see Figure 2C).
