# Supplementary material for: An improved strategy for CRISPR/Cas9 gene knockout and subsequent wildtype and mutant gene rescue
Source: PLoS One. 2020 Feb 13;15(2):e0228910. doi: 10.1371/journal.pone.0228910 (PMC7018052; doi:10.1371/journal.pone.0228910)
Supplement: S2 Fig — For each electropherogram, the wildtype (WT) sequence is aligned at the bottom along with gRNA sequence. In cell line GT5, four RhoA clones KO1, 3, 6 and 8 showed genomic sequences in the vicinity of gRNA5 region. Various alterations were observed at gRNA5 binding region. Clone KO1 had an A missing at the 17th nt of gRNA5, causing frameshift. Clone 3 was not a single clone, but a mixed one, possibly comprised of 2 clones, though Western blot showed it was truly RhoA KO (see Fig 2D). Clones KO6 and KO8 had 12 nt and 26 nt deleted at the 16th or -2nd nt of gRNA5 binding regions, respectively. Western blot showed both were truly RhoA KO (see Fig 2D). (DOCX) [file pone.0228910.s002.docx]

GT5 RhoA KO Single Clones


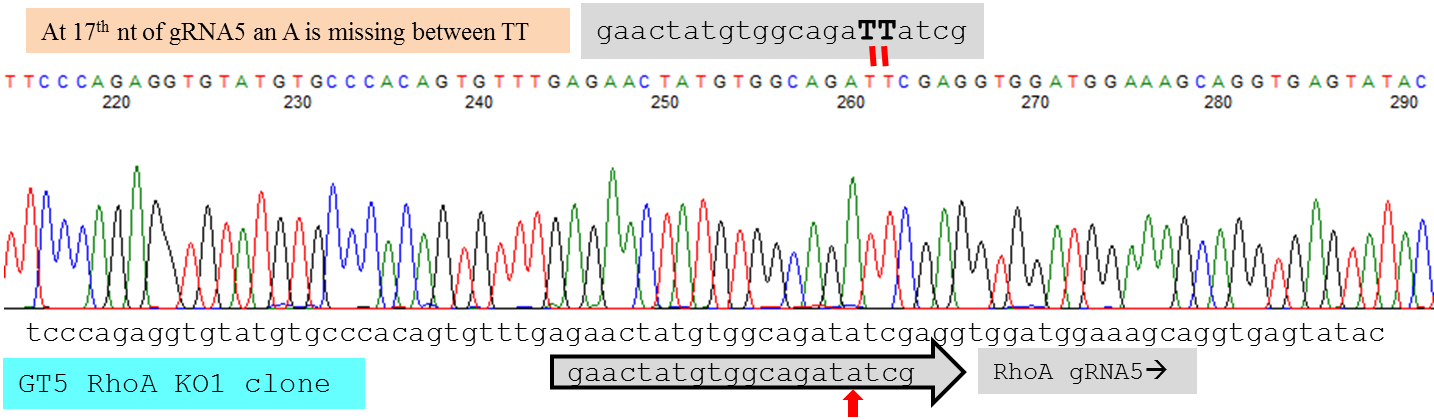


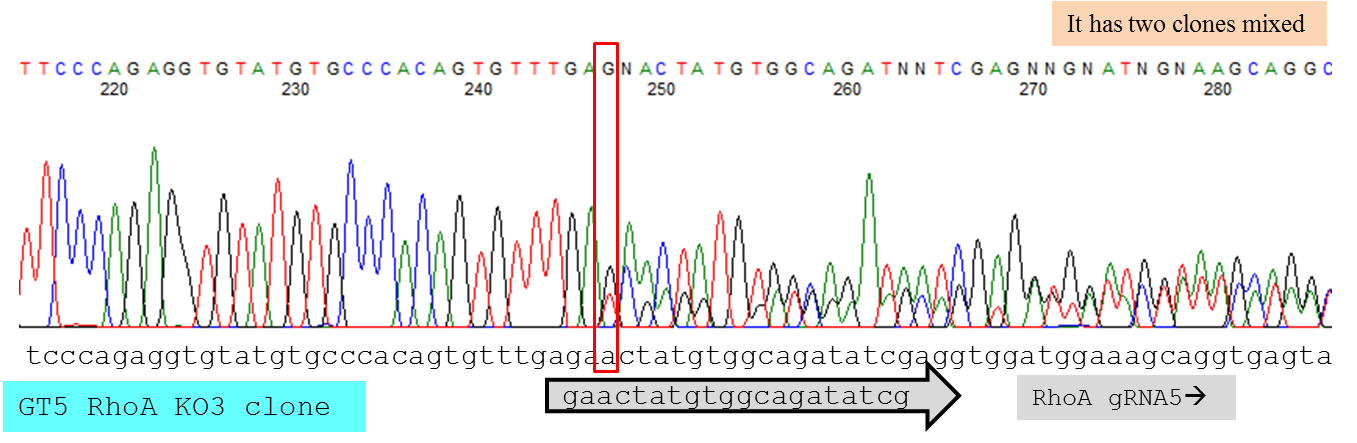


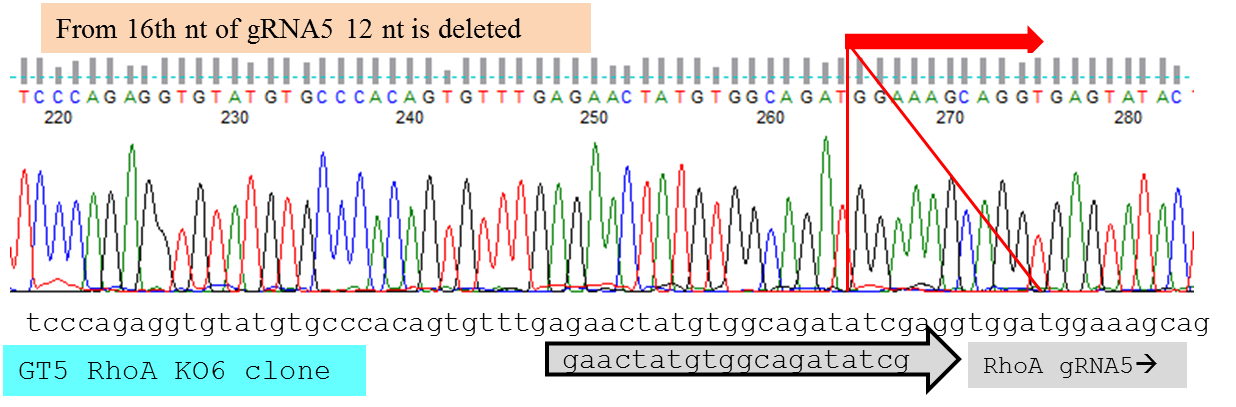


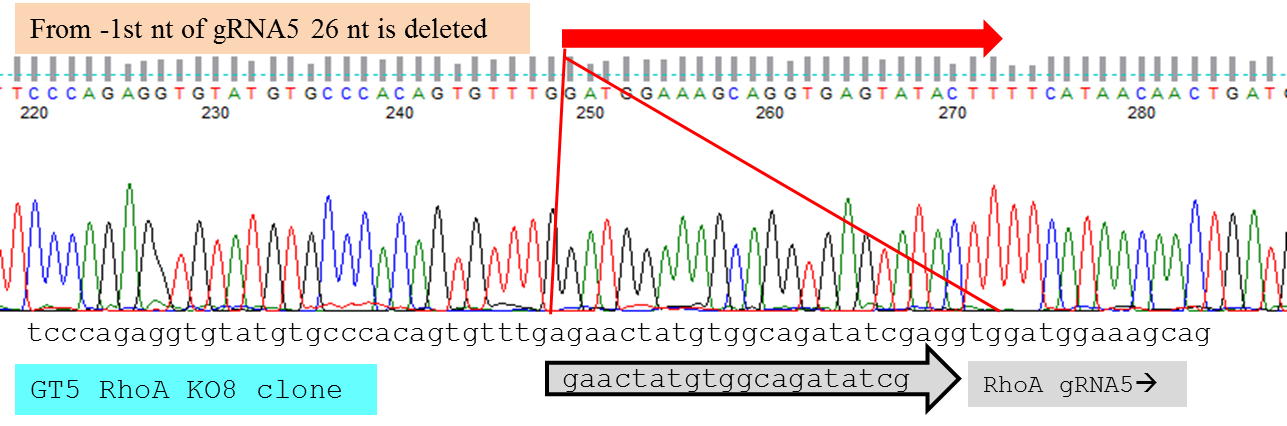


S2 Fig. Electropherograms of single clones of RhoA knockout (KO) from the GT5 cell line aligned with wildtype sequence. For each electropherogram, the wildtype (WT) sequence is aligned at the bottom along with gRNA sequence. In cell line GT5, four RhoA clones KO1, 3, 6 and 8 showed genomic sequences in the vicinity of gRNA5 region. Various alterations were observed at gRNA5 binding region. Clone KO1 had an A missing at the 17^th^ nt of gRNA5, causing frameshift. Clone 3 was not a single clone, but a mixed one, possibly comprised of 2 clones, though Western blot showed it was truly RhoA KO (see Figure 2D). Clones KO6 and KO8 had 12 nt and 26 nt deleted at the 16^th^ or -2^nd^ nt of gRNA5 binding regions, respectively. Western blot showed both were truly RhoA KO (see Figure 2D).
