# Supplementary material for: An improved strategy for CRISPR/Cas9 gene knockout and subsequent wildtype and mutant gene rescue
Source: PLoS One. 2020 Feb 13;15(2):e0228910. doi: 10.1371/journal.pone.0228910 (PMC7018052; doi:10.1371/journal.pone.0228910)
Supplement: S3 Fig — For each electropherogram, the wildtype (WT) sequence is aligned at the bottom along with gRNA sequence. In AGS cell line, Gal3 clones KO1, 3, 5, and 7 showed genomic sequences in the vicinity of gRNA1 region. Clones KO3 and KO5 had an extra T inserted at the 17th nt, causing frameshift. Clones KO1 and KO7 were not single clones, but mixed ones, though Western blot showed that they were truly RhoA KOs (see Fig 2F). (DOCX) [file pone.0228910.s003.docx]

AGS Gal3 KO Single Clones


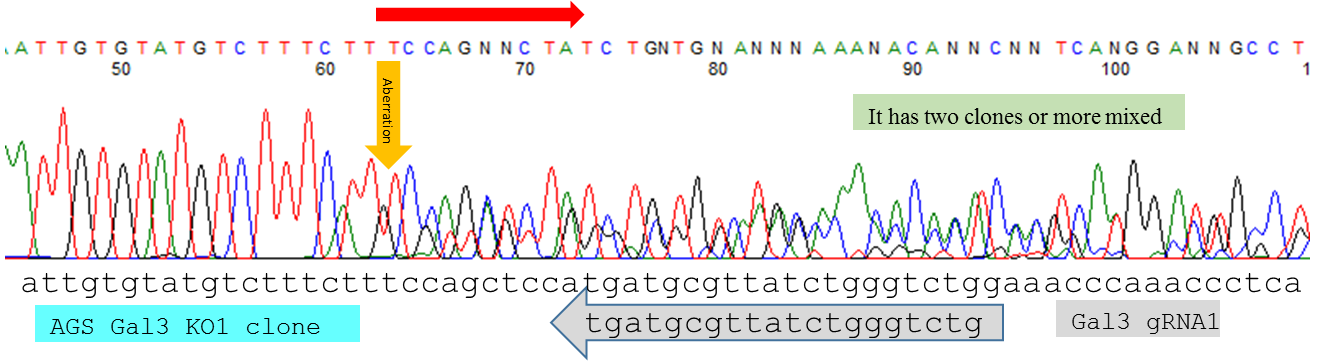


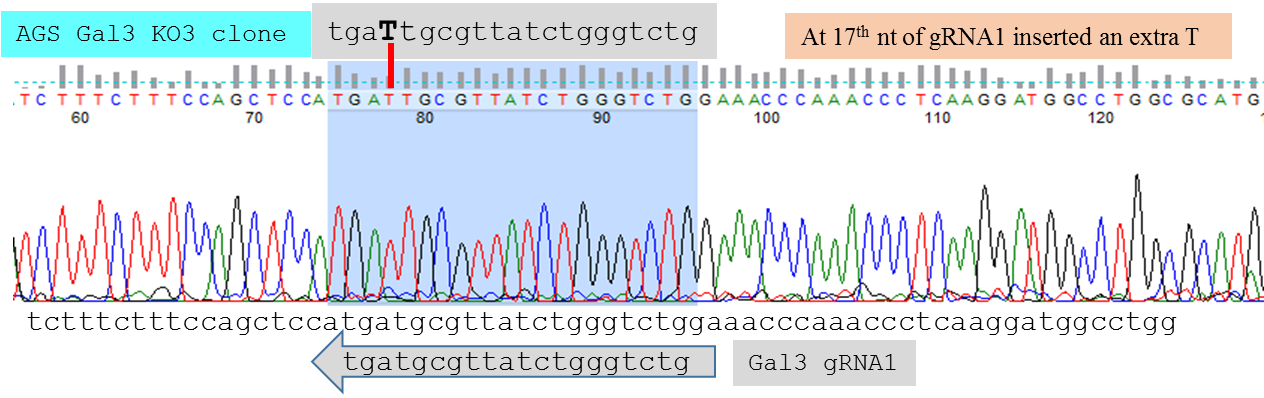


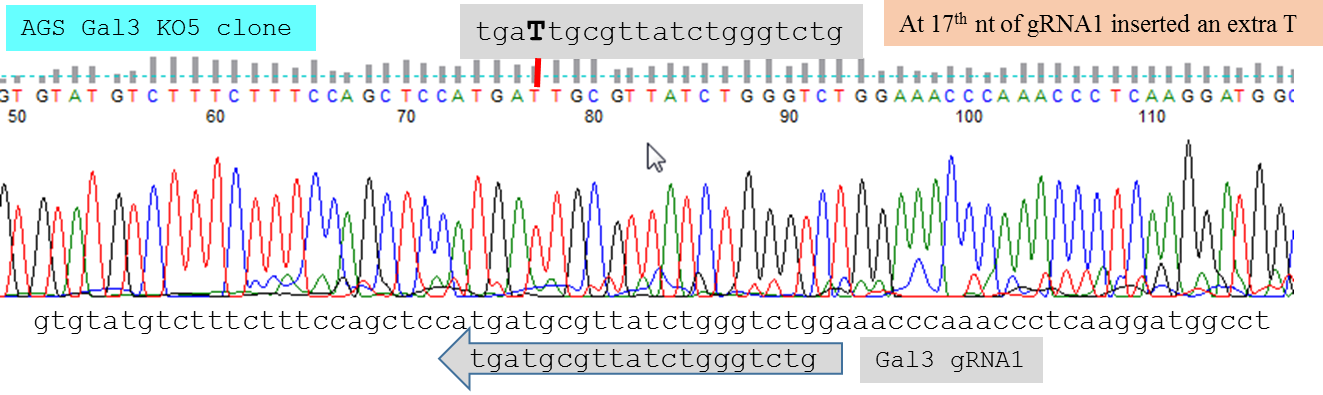


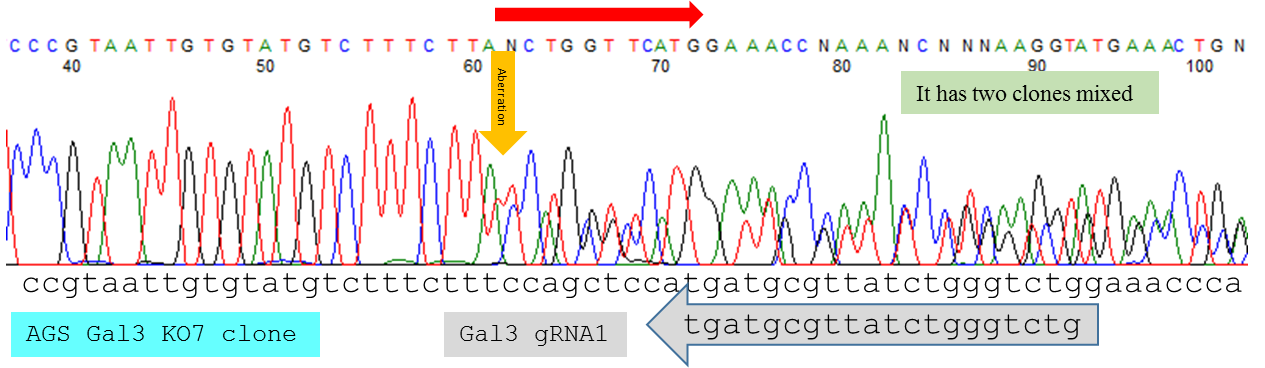


S3 Fig. Electropherograms of single clones of Gal3 knockout (KO) from the AGS cell line aligned with wildtype sequence. For each electropherogram, the wildtype (WT) sequence is aligned at the bottom along with gRNA sequence. In AGS cell line, Gal3 clones KO1, 3, 5, and 7 showed genomic sequences in the vicinity of gRNA1 region. Clones KO3 and KO5 had an extra T inserted at the 17^th^ nt, causing frameshift. Clones KO1 and KO7 were not single clones, but mixed ones, though Western blot showed that they were truly RhoA KOs (see Figure 2F).
