# Supplementary material for: An improved strategy for CRISPR/Cas9 gene knockout and subsequent wildtype and mutant gene rescue
Source: PLoS One. 2020 Feb 13;15(2):e0228910. doi: 10.1371/journal.pone.0228910 (PMC7018052; doi:10.1371/journal.pone.0228910)
Supplement: S4 Fig — For each electropherogram, the wildtype (WT) sequence is aligned at the bottom along with gRNA sequence. In cell line GT5, Gal3 clones KO3, 9, and 23 showed genomic sequences in the vicinity of gRNA1 region. Clone KO23 had an extra T/C inserted at the 17th nt, causing frameshift. Clones KO3 and KO9 were not single clone, but a mixed one, aberration began at 18th or 19 nt of gRNA binding sequence, though Western blot showed it was truly RhoA KO (see Fig 2G). (DOCX) [file pone.0228910.s004.docx]

GT5 Gal3 KO Single Clones


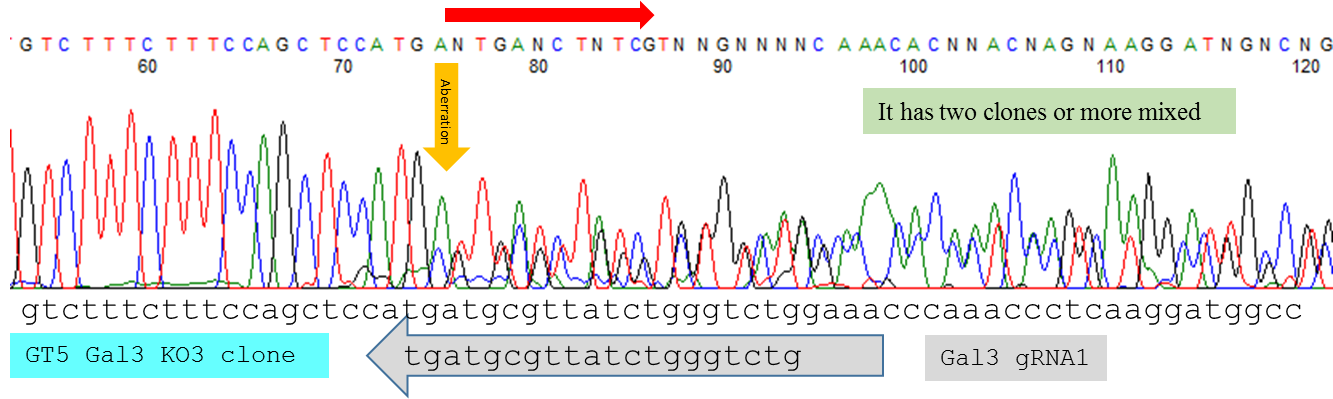


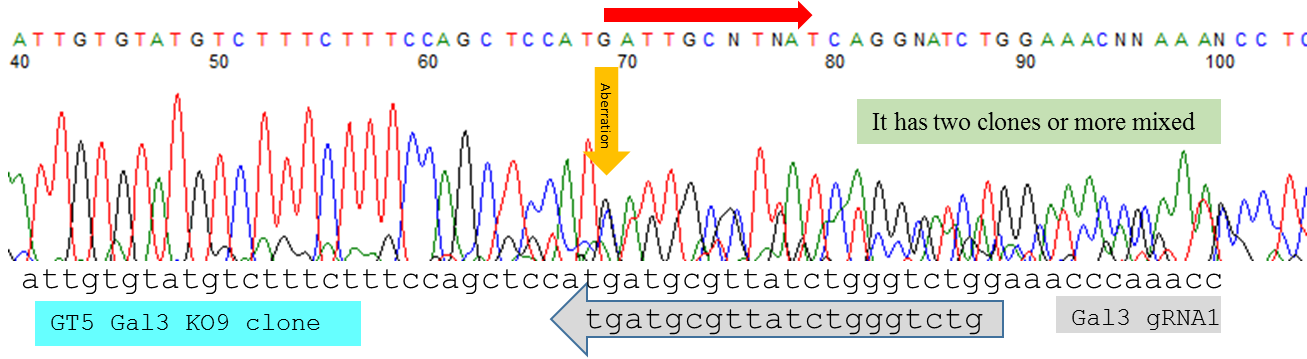


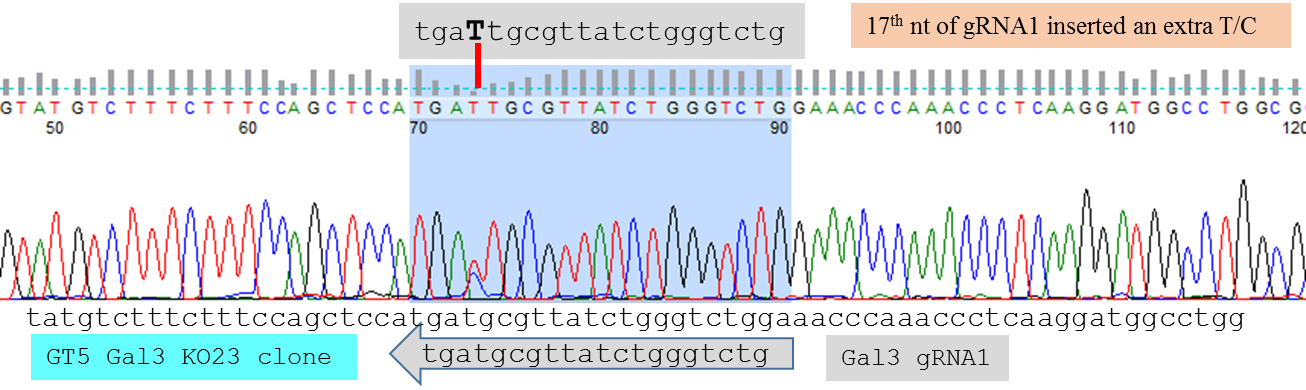


S4 Fig. Electropherograms of single clones of Gal3 knockout (KO) from the GT5 cell line aligned with wildtype sequence. For each electropherogram, the wildtype (WT) sequence is aligned at the bottom along with gRNA sequence. In cell line GT5, Gal3 clones KO3, 9, and 23 showed genomic sequences in the vicinity of gRNA1 region. Clone KO23 had an extra T/C inserted at the 17^th^ nt, causing frameshift. Clones KO3 and KO9 were not single clone, but a mixed one, aberration began at 18^th^ or 19 nt of gRNA binding sequence, though Western blot showed it was truly RhoA KO (see Figure 2G).
